# Supplementary material for: Pandora: nucleotide-resolution bacterial pan-genomics with reference graphs
Source: Genome Biol. 2021 Sep 14;22:267. doi: 10.1186/s13059-021-02473-1 (PMC8442373; doi:10.1186/s13059-021-02473-1)
Supplement: Supplementary file 2 — Additional file 2: Review history. [file 13059_2021_2473_MOESM2_ESM.docx]

**1^st^ round**

**Reviewer 1**

This paper from Colquhoun et al offers us one of the first serious attempts to genotype variants in the accessory genome of bacterial species. This is a challenging problem which, to my knowledge, has not been directly addressed before. Although graph genotyping methods have become available, they are targeted at eukaryotes, or even more specifically humans, and either do not work at all or perform poorly when applied to bacterial populations. Here, the authors combine their insights into how bacterial populations evolve with modern and creative genome informatics methods, and deliver a tool which I imagine will form the basis for this type of analysis in many future studies.

The manuscript and associated analyses clearly represent the culmination of a lot of thought and work from many people. The paper clearly describes the issue at hand, implements a solution which outperforms other methods in its targeted domain of accessory SNPs, and carries out a very careful benchmarking of performance which is just as technically impressive as the method itself. While there may eventually be other ways to approach this problem, this method and paper will introduce the field to this new problem, and I am sure the authors will continue to build and extend this work.

I was able to install and run the tool on some of my own test data, though after some hurdles (noted below). I would also commend the authors on the reproducibility of the work and code, which is amongst the best I have seen.

Currently, the manuscript reads mostly as a detailed technical report describing pandora's design and performance as a variant caller, versus other comparable methods. Readers from a more applied background wondering how they might benefit from the tool will not find direct applications of where improved variant calls may be used downstream. However, I believe the authors have made the right choice to focus their first manuscript this way, especially given the technical detail here, which will be useful for future development efforts. The manuscript is long and detailed, and I do not think further substantive analyses are necessary.

Overall, I was excited to read this paper, and I think it makes an important and imaginative contribution to a challenging topic. I am sure the tools will be used widely by a broad range of researchers. I have only minor comments, many of which are suggestions the authors can use their discretion as to whether to explore further.

John Lees

Specific comments:

- I think the authors should carefully consider the downstream applications for which pandora will be most useful, and state exactly what advantages/disadvantages would be expected versus 'competing' cutting edge methods. This is currently addressed in the discussion, but some mention of uses should probably go in the introduction. Some thoughts which may supplement the discussion:

- GWAS: are accessory gene SNPs any better than using k-mers or unitigs, and why? I think the interpretation of results will likely be easier, but do the authors have further thoughts?

- Epidemiology: comparison is made with wgMLST schemes, but the specifics of this comparison weren't clear to me (does pandora give an 'allele' to each sample and gene in its output?). Alignment-free methods for wgMLST are also available - how might pandora compare to them?

- Phylogenetics: I can certainly see how pandora may improve detailed phylogenetics versus a core genome alignment. How would missing SNPs be dealt with in this kind of application? Would a partitioned analysis be advisable?

- Selection/adaptation: I am excited to use pandora to calculate dN/dS of accessory SNPs. I cannot think of a way to do this with alignment-free methods. If the authors agree, perhaps this is worth mentioning?

- The benchmarking of SNP calls is a challenging problem in itself, and I think the authors have done a thorough job here. Could the authors comment whether the benchmarking process is likely to suffer from false positives/negatives due to its complexity, and if so whether it's possible to estimate how much effect on results this may have?

- The authors compare pandora with alignment versus the closest sequence in RefSeq. Would aligning to an assembly from within the same sequence cluster also be viable, especially where RefSeq is not well-sampled? I have seen this used a few times before. Can the authors describe the advantages of pandora over this approach?

- Is it possible to predict (using either a simple Monte Carlo method, or other out-of-the-box bacterial population simulator) how many SNPs are likely to be missed for the different species noted, if the accessory genome was ignored? This could be a nice addition to figure 1, if easily tractable.

- While I think figures 5-8 are a very honest account of the method's strengths and weaknesses, they are all fairly complex, and require careful reading of the text to follow. Rather than starting with the methylation calling figure, I wondered whether the authors could design a figure which clearly shows the major advantage of pandora. I thought this was probably shown by the left 25% of the panels in figure 7, which are presently a little dense and hidden.

- I am not particularly familiar with nanopore sequencing, and had trouble understanding the sections relating to this technique. It seemed odd to start the results/fig 5 comparing methylation-aware basecalling of these long reads with other methods in the de novo assembly stage - which is certainly not the main thrust of the paper. Moreover, it was not immediately clear to me even what relation nanopore basecallers had with pandora, or how results would be comparable. 'methylation-aware basecalling' was not defined, or why it would be expected to improve recall. My suggestion would be to move this section to the supplementary material, which would also help reduce the length of the results.

- I felt like the order of paragraphs in the background caused the narrative to jump around, and wonder whether the structure might be rethought. Perhaps the second paragraph on genes should go in the results.

- Describing the population as mosaics felt like a very 'human genetics' way of describing the problem rather than bacterial genomics, and might lose some readers. I didn't really feel like the idea of mosaics was returned to, especially when de novo assembly is used to find new parts of sequence not in the reference. Sources of recombination aren't mentioned here - would a prophage integrating into a reference be legitimately be described as a mosaic?

- I think it would really help to have a more intuitive explanation of what (w,k)-minmizers are in the main text, and why they were used over other indexing methods. The current description is fairly opaque to those who have not come across them before.

- At the beginning of the methods, I think it would be useful to add an overview of the steps for calling variants as in the snakemake pipeline (or show the DAG), and which of the author's software packages/commands perform each step.

- Some investigation or discussion of the choice of atomic unit used to build each graph would be helpful. Taking it to the extremes: why not align the whole genome? Why not align small regions within genes? What would be the advantages and disadvantages of taking operons or groups of genes together?

- In figure 2, it should be made clear that the pangenome matrix is as defined by the input clusters (in this case panX), and pandora provides presence/absence wrt this reference, it doesn't cluster genes itself.

- Was gubbins run across E. coli phylogroups to remove recombination? If so, did it converge, and remove a reasonable proportion of the alignment? It can be problematic with genetically distant input.

- In the computational performance the methods were compared with different numbers of cores, which makes it difficult to compare resource use on an even footing.

Software comments:

- The relation between make_prg and pandora was not entirely clear to me, and I couldn't confirm whether pandora use make_prg's output. Perhaps combining them in a single repository would be sensible?

- I think it would be sensible to put make_prg on either/both PyPI and/or conda. Currently the dependencies are unmanaged, and it installs into the global environment by default.

- I used the docker image to run pandora. Though the command to pull it is given, no further information on how to use this image and share data with it was listed. It took a little time to work this out. I would recommend adding instructions on how to then use the image to the README.

Additionally, what would you recommend when docker is not an option for pandora (e.g. on HPC systems)?

- It was difficult to know whether I should be using an unaligned fasta or MSA for each of the make_prg and pandora steps. It would be useful to highlight this. pandora gives a slightly uninformative error message if you use an MSA as input.

- When running 'pandora map' does this only accept a single read file? What if reads are in two forward/reverse files? I concatenated my files, but might be worth addressing this in the docs.

- When I ran 'pandora map' it looked successful, but I didn't get any variants in the VCF output (using -I --genotype). Could this be expected?

- The authors may wish to investigate pybind11 for their planned python->C++ conversion, to keep the useful python code and not reimplement everything (command line args etc) in C++. I have previously found this package a joy to use.

- Are Ns disallowed in make_prg input? I got an EncodeError using an alignment with Ns.

Small edits:

- In the abstract: 'The pragmatic option' -> 'a commonly used pragmatic option'

- p12 l15: 'key deliverable' -> 'main advantage'

- In the discussion: 'not a production tool'. Can the authors expand on exactly what they mean by this? Should others not use this software? My impression is that it would be good for others to start using pandora for testing, but not yet incorporate it into their pipelines as standard.

- Conclusions: 'to our knowledge, the first solution'. I would advise against claims such as this - the novelty of this work stands on its own.

- Methods p22 l31 'log-likelihood-inspired' - please explain what these means and why it was used.

- I could not distinguish between the colours in fig1a - could an alternative diverging scheme be used?

- In fig1b I was unable to easily match the colours of genes across the genomes. Highlighting syntenic blocks of genes between each sample with shaded blocks (as in ACT or genomeribbon)

**Reviewer 2**

The authors describe a novel method, pandora, enabling variant calling using a set of reference genomes stored in a graph data structure. The manuscript is well written and describes many smart features of pandora together with evaluations comparing pandora to single reference variant callers.

In general I think this is an interesting and impressive manuscript. I have, however, a few remarks:

- the main argument for pandora described in the manuscript is that detecting SNPs in the core genome is not enough. However, the authors do not provide much proof/examples for how SNPs in the accessory genome - once identified - can be used to enhance our understanding of these genomes. As this is not clear to me through the manuscript, the argument loses its power somehow.

At least it would have be great to see how the accessory genome SNPs identified in the E.coli dataset compare to the core genome phylogeny of these strains.

- what is the limit on the number of bacterial reference genomes pandora can handle? How do I select an appropriate set of reference genomes? I was also wondering if the result influenced by the diversity of chosen reference genomes?

- The comparisons with single-reference tools seem to have been carefully designed and are certainly not easy to do, however the presented comparison in the paper is also not easy to understand. Figure 6 shows "precision" of the evaluated methods, however I could not find a definition of precision in the text.

- While I understand that graph-based variant calling is hit and new, there are already a few tools out there that address the problem (VG toolkit? GraphTyper? Paragraph? PanGenie and Giraffe (admittedly these two only appeared in 2020). It would have been useful to see a direct comparison with other graph based methods to understand the advantages of pandora, or at least add a paragraph discussing these other methods in comparison to pandora.

- I think the section on Methylation-aware basecalling is disrupting the evaluation as it discusses the quality of the read data instead of the actual comparison laid out in the previous section, maybe move this into appendix?

- Figure 7 seems to suggest that while pandora is able to recover more SNPs in the accessory genome, it also misses SNPs in the core genome (e.g. present in 19 or 20 genomes in the sample) that are detected by snippy and samtools. This should be addressed in the discussion, maybe the authors want to add a suggestion on when to use pandora and when to rely on snippy?

- All analyses in this paper seem to be well documented and reproducible in snakemake pipelines (awesome!), so I tried to run some code myself. I tried installing pandora from source code on my server (no docker allowed unfortunately), but ran into errors compiling gatb (lib and boost installed though). It would be great if there were a few hints on common compile problems on the GitHub page (if known). I was able to install pandora on my laptop through docker without problems. I then wanted to try pandora on a set of my genomes, but realised that the tool building the reference graph only takes MSAs as input. It would be great if this could be extended to include the alignment step.

I went back to use the hands-on toy example provided on the website and that seemed to work once I figured out how to use docker properly. I personally would prefer to use pandora as a source code installation.

**Author response**

Reviewer #1

This paper from Colquhoun et al offers us one of the first serious attempts to genotype

variants in the accessory genome of bacterial species. This is a challenging problem which,

to my knowledge, has not been directly addressed before. Although graph genotyping

methods have become available, they are targeted at eukaryotes, or even more specifically

humans, and either do not work at all or perform poorly when applied to bacterial

populations. Here, the authors combine their insights into how bacterial populations evolve

with modern and creative genome informatics methods, and deliver a tool which I imagine

will form the basis for this type of analysis in many future studies.

The manuscript and associated analyses clearly represent the culmination of a lot of thought

and work from many people. The paper clearly describes the issue at hand, implements a

solution which outperforms other methods in its targeted domain of accessory SNPs, and

carries out a very careful benchmarking of performance which is just as technically

impressive as the method itself. While there may eventually be other ways to approach this

problem, this method and paper will introduce the field to this new problem, and I am sure

the authors will continue to build and extend this work.

I was able to install and run the tool on some of my own test data, though after some hurdles

(noted below). I would also commend the authors on the reproducibility of the work and

code, which is amongst the best I have seen.

Currently, the manuscript reads mostly as a detailed technical report describing pandora's

design and performance as a variant caller, versus other comparable methods. Readers

from a more applied background wondering how they might benefit from the tool will not find

direct applications of where improved variant calls may be used downstream. However, I

believe the authors have made the right choice to focus their first manuscript this way,

especially given the technical detail here, which will be useful for future development efforts.

The manuscript is long and detailed, and I do not think further substantive analyses are

necessary.

Overall, I was excited to read this paper, and I think it makes an important and imaginative

contribution to a challenging topic. I am sure the tools will be used widely by a broad range

of researchers. I have only minor comments, many of which are suggestions the authors can

use their discretion as to whether to explore further.

John Lees

These positive comments are very much appreciated, thank you!

Specific comments:

- I think the authors should carefully consider the downstream applications for which pandora

will be most useful, and state exactly what advantages/disadvantages would be expected

versus 'competing' cutting edge methods. This is currently addressed in the discussion, but

some mention of uses should probably go in the introduction.

We agree and have added some text to the introduction on line 60

Some thoughts which may supplement the discussion:

- GWAS: are accessory gene SNPs any better than using k-mers or unitigs, and why? I think

the interpretation of results will likely be easier, but do the authors have further thoughts?

In our opinion, the primary advantage for GWAS is interpretation, as our method explicitly

places a variant in the context of a gene and we have clarified this on line 577, where we say

*“We finish with potential applications of pandora. First, the PanRG should provide a more*

*interpretable substrate for pan-genome-wide Genome-Wide Association Studies, as current*

*methods are forced to either ignore the accessory genome or reduce it to k-mers or unitigs*

*[59–61] abstracted from their wider context.”*

There is also a subtle technical benefit, as pandora genotypes SNPs based on coverage

information from mapping entire reads - coverage of minimizing k-mers in the pandora graph

is not registered unless the read is confidently mapped to the gene - this significantly

reduces the number of false hits of k-mers. By contrast, k-mer approaches by necessity

conflate different instances of a repetitive k-mer.

- Epidemiology: comparison is made with wgMLST schemes, but the specifics of this

comparison weren't clear to me (does pandora give an 'allele' to each sample and gene in its

output?). Alignment-free methods for wgMLST are also available - how might pandora

compare to them?

Pandora is like wgMLST in that it restricts analysis to a (large) set of loci for which it has a

catalogue of previously seen variation, and this is used as a substrate for (geno)typing new

sequences. However unlike wgMLST, pandora will allow noisy recombinants of known

alleles. We had included this comparison largely in order to frame what we do in a way that

is more comfortable to a group of potential users, not because we think pandora should

replace wgMLST or other methods of determining wgMLST type. We have decided to

remove the comment to avoid confusion.

- Phylogenetics: I can certainly see how pandora may improve detailed phylogenetics versus

a core genome alignment. How would missing SNPs be dealt with in this kind of application?

Would a partitioned analysis be advisable?

A partitioned analysis is exactly what we would recommend, with the VCF restricted to loci in

the relative core genome of any subset of samples. (This would allow analyses of different

subsets without having to choose a new reference and remap and recall variants.) In this

situation missing SNPs would be no more of a problem than with any other tool that

generates a VCF.

We have added the following paragraph to the discussion, line 565.

“*For bacterial genomes, genotype calls are often used to perform phylogenetic analyses. By*

*detecting accessory variation, three things become possible. First, pragmatically, one can*

*choose clusters of similar genomes based on the cohort-wide core genome, and then by*

*restricting the pandora VCF to genes present in each cluster, re-analyse based on the*

*cluster-specific core genome. Normally this would require choosing a cluster-specific*

*reference, remapping reads and re-running a variant caller, but with pandora all of the*

*necessary data is provided in one step. Secondly, the accessory SNPs provide an extra level*

*of resolution when comparing samples which are very close on a core-genome tree, which*

*may be useful. Finally, one cannot represent all pan-genome variation in a phylogeny as the*

*evolutionary history is fundamentally not compatible with a simple vertical-inheritance model.*

*However the pandora output would make ideal material on which to build and test population*

*genetic models.*”

- Selection/adaptation: I am excited to use pandora to calculate dN/dS of accessory SNPs. I

cannot think of a way to do this with alignment-free methods. If the authors agree, perhaps

this is worth mentioning?

We too are excited about applying pandora to selection (eg via dN/dS) in the accessory

genome, and have added a comment to this effect to the paper on lines 61 and 580.

However, we do not expand further on this comment since, when looking at dN/dS, you need

to control for the time to the most common ancestor, which is different in accessory genes

compared with the core genome (see Rocha et al.

https://pubmed.ncbi.nlm.nih.gov/16239014/). Thus although we see this as a promising

avenue, the path is not completely clear.

- The benchmarking of SNP calls is a challenging problem in itself, and I think the authors

have done a thorough job here. Could the authors comment whether the benchmarking

process is likely to suffer from false positives/negatives due to its complexity, and if so

whether it's possible to estimate how much effect on results this may have?

We think there are fundamentally two ways in which a benchmarking process might

mis-count true or false calls. We address the easier issue first: any software process is likely

to have errors in proportion to the number of lines of code, and our benchmarking pipeline is

not small (roughly 5000 lines of code). We do not think this will contribute an appreciable

number of errors, because we designed unit tests into the code from the start (roughly 6000

lines of test code), and have extensively manually checked all of these results. Unit tests

are, in theory, the escape route from errors-per-line-of-code. Secondly, it is undeniable that

our benchmarking conceptual approach is relatively complicated. That complication is

inescapable as soon as you want to find all SNPs between bacterial genomes, for the

reasons outlined in our paper - no single coordinate system will work. We followed a similar

approach to that taken by the Genome-In-A-Bottle consortium

(https://www.nature.com/articles/nbt0715-675a), who defined a conservative golden truth set

for recall evaluations, knowing that it missed some true variants, but seeking variants that

were definitely true. Our approach is similarly conservative, finding SNPs between pairs of

genomes, and filtering carefully to remove any SNPs in loci with paralogs. This benchmark

will not evaluate the ability to call SNPs in difficult repeat regions, or in genes with

duplicates. It is also likely that the truth set of SNPs will be depleted of SNPs in short

(100bp) genes which occur in multiple contexts, as our “probes” which define SNPs by their

flanking sequence, would end up overlapping those multiple contexts. Nevertheless we

believe it is a careful and reliable method for finding true variants and evaluating their

detection.

Having said all this, we must confess that during the revision process, we did find an

edge-case bug in the evaluation framework. You may remember that in order to measure

precision, for each VCF record, we take the called allele and apply it to the reference

genome, and then create a “probe” consisting of the allele, and flanking sequence on either

side. The edge case was as follows: when a variant occurred near the end of a contig in the

reference genome used for the VCF, and when in addition the probe mapped to the truth

genome in the reverse complement direction, we checked the correctness of the wrong

allele bases (they were “off by reverse complement”). This error happened essentially never

for the single-reference callers, as they used standard reference genomes made of 1 contig.

However, it happened very often for pandora, as each locus was its own contig in the VCF

reference, so variants were frequently near the end of a contig. Fixing this bug reduced the

pandora error rate from 0.6% to 0.2% (and did not affect the error rates of the other tools).

- The authors compare pandora with alignment versus the closest sequence in RefSeq.

Would aligning to an assembly from within the same sequence cluster also be viable,

especially where RefSeq is not well-sampled? I have seen this used a few times before. Can

the authors describe the advantages of pandora over this approach?

In the case of our small benchmark, all 20 of our samples are relatively close to a genome in

RefSeq (see Figure 4), so this question is somewhat redundant. However, in general, as the

reviewer suggests, RefSeq will not be well sampled, and one could simply use an assembly

of one of the samples in a cluster as a reference. This would work just as well as using a

nearby external reference - i.e. it would work for closely related samples, but not so well for

those further away (as shown in Figure 7 on line 395).

Looking at this in terms of where Pandora is or is not useful - Pandora really offers an

advantage where the dataset in question includes diversity. Single-reference methods work

best when there is little diversity, such as in an outbreak. In a diverse set, pandora allows the

user to spot variation within accessory genes shared across highly diverged genomes. There

is also an unexpected benefit. Typically to study a diverse set with standard methods, one

will first use an arbitrary reference to cluster genomes, and then re-run the analysis for each

cluster using a cluster-specific reference. Pandora allows you to run just once, and then

analyse each cluster by restricting to its own core genome.

- Is it possible to predict (using either a simple Monte Carlo method, or other out-of-the-box

bacterial population simulator) how many SNPs are likely to be missed for the different

species noted, if the accessory genome was ignored? This could be a nice addition to figure

1, if easily tractable.

We agree that this would be interesting for the user, but decided that it was not easily

tractable within the available space in this paper. For any given U-shaped gene-frequency

curve, if we assumed all genes were independently distributed (i.e. no population structure

and freely recombining), we could use an average mutation rate to estimate the number of

accessory SNPs missed by a random reference genome. However the assumption of no

structure is strong, and we felt this would be better addressed in another paper, combining

the modelling with empirical measures of this for various species.

- While I think figures 5-8 are a very honest account of the method's strengths and

weaknesses, they are all fairly complex, and require careful reading of the text to follow.

Rather than starting with the methylation calling figure, I wondered whether the authors

could design a figure which clearly shows the major advantage of pandora. I thought this

was probably shown by the left 25% of the panels in figure 7, which are presently a little

dense and hidden.

We thank the reviewer for helping us see this; we agree and have moved Figure 5 to

Supplementary Materials, and the associated text to the methods. We have switched the

sections corresponding to Figures 6 and 7 to move the primary advantage of accessory

variation calling earlier in the text and have restricted this figure to 2 panels for clarity

(moving the remaining 2 panels to Supplementary Materials). Whilst we agree that the 4

panels in Figure 6 are dense, we feel that the large difference between Nanopore and

Illumina results mean that all panels are necessary to convey the story. However we do

feel the new text is easier to follow.

- I am not particularly familiar with nanopore sequencing, and had trouble understanding the

sections relating to this technique. It seemed odd to start the results/fig 5 comparing

methylation-aware basecalling of these long reads with other methods in the de novo

assembly stage - which is certainly not the main thrust of the paper. Moreover, it was not

immediately clear to me even what relation nanopore basecallers had with pandora, or how

results would be comparable. 'methylation-aware basecalling' was not defined, or why it

would be expected to improve recall. My suggestion would be to move this section to the

supplementary material, which would also help reduce the length of the results.

We agree and have moved the figure to Supplementary Materials. Pandora was initially

developed as a Nanopore genotyping tool, but along the way we realised our methods were

generalizable to solve the larger problem of accessory genotyping with any sort of read data.

The results and focus of this paper are much clearer without this in the results.

Just to follow on, we would remark that although nanopore sequencing has advantages

(long reads), it has a major drawback that is often sold as an advantage. The electrical

signals which are measured as the DNA passes through a pore, are affected by more than

just the strict DNA sequence. Other factors - especially DNA damage and methylation state -

affect the electrical signal, and therefore the basecalling. Thus nanopore sequencing is error

prone with “native” DNA, and gives cleaner results after PCR-based library preparation

(which effectively removes DNA damage). Most importantly, there appears to be an accuracy

ceiling for nanopore reads - DNA methylation state causes errors in basecalling. This can be

fixed by training the neural network used to do base-calling, giving it information on the

sequence motifs associated with methylation - however this requires species-specific

training of the basecaller, and for the relevant motifs to be known. Thus, unlike Illumina

technology, where a universal basecaller exists, and sequences from different experiments

stored at the EBI/NCBI can be compared, this is not true for nanopore. The error-profile of

nanopore depends on the basecaller version, the species being sequenced, and whether the

methylation motifs for the species are known and have been used to train the basecaller. In

our case, E coli is heavily studied, and has been used in training the standard basecaller,

guppy, so we evaluated the benefit of turning this aspect on. It did indeed make a significant

difference. But we 100% agree this is a technical detail about how the nanopore data is

processed, and does not deserve to be the centre of attention in our paper.

- I felt like the order of paragraphs in the background caused the narrative to jump around,

and wonder whether the structure might be rethought. Perhaps the second paragraph on

genes should go in the results.

We have cut down and simplified this paragraph for clarity, removing specifics to *E. coli*. We

do feel that we need to make the case that in the context of the bacterial pangenome where

most genes are rare, single reference methods are flawed.

- Describing the population as mosaics felt like a very 'human genetics' way of describing the

problem rather than bacterial genomics, and might lose some readers. I didn't really feel like

the idea of mosaics was returned to, especially when de novo assembly is used to find new

parts of sequence not in the reference. Sources of recombination aren't mentioned here -

would a prophage integrating into a reference be legitimately be described as a mosaic?

Thank you for highlighting this. We have now added a paragraph to the discussion at line

524 on how types of recombination lead to different levels of genomic mosaicism, and how

this should motivate the choice of atomic unit used with our method. We include this

paragraph here:

*“With pandora, we break the genome into atomic units which may reorder freely between*

*samples, but within which the degree of variation is more constrained. This approach is*

*directly motivated by our knowledge of the mechanisms underlying genomic flexibility in*

*bacteria. The breadth of diversity we see in these bacteria arises primarily as a result of*

*horizontally acquired DNA which is incorporated into the genome by different forms of*

*recombination. Homologous recombination between closely related sequences may result in*

*allele conversion and in some species contributes as many nucleotide changes as point*

*mutation [52]. As a result, locally we expect sequences to look like mosaics of each other,*

*possibly with additional novel mutations. Genes are acquired (and lost) as a result of*

*homologous or site-specific recombination and at hotspots [53,54]. The dynamics of this are*

*organised [30], and result in global genome mosaicism. The choice of atomic unit used to*

*build each local graph should again be motivated by this underlying biology. A locus should*

*be large enough for its presence and sequence to be useful independently of other graphs,*

*but small enough as to be typically inherited as an entire unit. Biologically speaking, genes*

*fulfil this requirement and there already exist a plethora of tools designed to extract and align*

*genes (and intergenic regions) in a set of bacterial genomes (prokka [55], panaroo [56],*

*roary [57], panX [33], piggy [34]). Operons or groups of genes which co-occur contiguously*

*might also make a good choice, although isolating a set of reference sequences for these*

*regions would be more of a challenge.”*

Bacterial genomes can be thought of as mosaics on two levels - on a whole genome level

they are mosaics with units of sequence often (but not always) gained and lost together e.g.

Figure 9 *Touchon et al* 2009 “Organised Genome Dynamics in the *Escherichia coli* Species

Results in Highly Diverse Adaptive Paths”. Our methods explicitly assume that each local

graph represents such a unit and that inferred genomes are therefore mosaics of the

reference panel of genomes. The organised nature of recombination and genome dynamics

within bacteria is the very reason we can do this. With respect to our method, a prophage

integrating into the genome would still be a legitimate mosaic. Locally within a reference

graph, any path can be thought of as a mosaic of the reference sequences used to construct

the graph. This level of mosaicism does indeed have strong parallels with human genetics

and is the reason the inferred local path is referred to as a *mosaic path*.

- I think it would really help to have a more intuitive explanation of what (w,k)-minmizers are

in the main text, and why they were used over other indexing methods. The current

description is fairly opaque to those who have not come across them before.

We have added an informal description and motivation to line 155.

- At the beginning of the methods, I think it would be useful to add an overview of the steps

for calling variants as in the snakemake pipeline (or show the DAG), and which of the

author's software packages/commands perform each step.

We have improved the interface for genotyping with *de novo* discovery so that it only uses

single *pandora* and *make_prg* commands, as opposed to requiring in some places 1

command per local graph. The user can now replicate the same *pandora* execution workflow

by running only a few command lines:

make_prg from_msa

pandora index (on the original PanRG)

pandora discover

make_prg update

pandora index (on the updated PanRG)

pandora compare

We now also provide a self-contained sample example on how to run *pandora* and

*make_prg* and a description of the output at

https://github.com/rmcolq/pandora/tree/master/example . This example mimics how we use

*pandora* and *make_prg* in our evaluation in the paper. Although we use a pipeline manager

in our evaluation workflow for reproducibility, with these improvements the current *pandora*

workflow is very close to what is described in Figure 2, thus we chose to not show the

pipeline DAG as it would be mostly redundant.

- Some investigation or discussion of the choice of atomic unit used to build each graph

would be helpful. Taking it to the extremes: why not align the whole genome? Why not align

small regions within genes? What would be the advantages and disadvantages of taking

operons or groups of genes together?

This topic is related to discussion of types of recombination and mosaicism and we have

combined them within a new paragraph in the discussion section at line 524. These specific

questions have mostly been answered in the revised text, but in particular:

- Whole genome alignment of a diverse bacterial species does not scale well, but

more importantly it would result in a reference graph that requires/contains many

cycles due to rearrangement between genomes in the reference panel. Other

graph-genome tools espouse this approach on the grounds that it encodes the

full data, but then have to build huge machinery and implement many heuristics,

in order to cope with the cycles. *Pandora* does not allow cycles within a local

graph.

- Pandora detects loci by spotting seed k-mers (so-called (w,k)-minimizers) -

typically with k=15. Nanopore reads are error-prone, and so the probability of a

k-mer being correctly present in a read is not high. As a result, pandora suffers

from increased false negatives (failing to detect a gene/locus) when the locus is

short - i.e. under 200bp (Supplementary Figure 6). Thus graphs representing very

small regions within a gene would suffer loss of sensitivity, and we would not

recommend it.

- Graphs representing longer regions such as operons or groups of genes

together would make it easier to put variants into context. Collecting together a

panel of reference sequences to align might be more of a challenge. The

disadvantage is increased likelihood that a sample would contain only part of that

locus in a different context. In such a situation, pandora would either miss the

locus, or erroneously infer the whole operon/region was present in the sample.

- In figure 2, it should be made clear that the pangenome matrix is as defined by the input

clusters (in this case panX), and pandora provides presence/absence wrt this reference, it

doesn't cluster genes itself.

Agreed - we have corrected this.

- Was gubbins run across E. coli phylogroups to remove recombination? If so, did it

converge, and remove a reasonable proportion of the alignment? It can be problematic with

genetically distant input.

Gubbins was only run as part of the process to construct a phylogenetic tree for the manual

selection of a set of comparison references (used by the single-reference genotypers). All

other alignment was constructed pairwise.

- In the computational performance the methods were compared with different numbers of

cores, which makes it difficult to compare resource use on an even footing.

We agree and apologise. This paragraph has been rewritten and performance is now

reported as follows (see section “Computational performance”, line 469):

*“We report here the CPU time and maximum RAM consumed by the evaluated tools. All of*

*the single-reference tools analysed isolates independently, whereas pandora has a*

*subsequent joint analysis step to compare them all; we therefore compare the end-to-end*

*performance of pandora analysing all 20 samples against the mean performance of each*

*single-reference tool (summing all 20 samples, and then averaging over the different*

*reference genomes). In short, pandora took 9.2 CPU hours to analyse the 20 isolates with*

*Illumina data while snippy and SAMtools both took 0.4 CPU hours. With Nanopore data,*

*pandora took 16.4 CPU hours, which is slower than medaka (0.7 CPU hours), but faster than*

*nanopolish (84 CPU hours). In terms of memory usage, for the Illumina data, pandora used*

*a maximum of 13.4 GB of RAM, compared with snippy (3.2 GB), and SAMtools (1.0 GB),*

*whereas for the Nanopore data, pandora used a maximum of 15.7 GB of RAM, compared*

*with medaka (5.9 GB) and nanopolish (10.4 GB).”*

Software comments:

- The relation between make_prg and pandora was not entirely clear to me, and I couldn't

confirm whether pandora use make_prg's output. Perhaps combining them in a single

repository would be sensible?

This is a fair question. The reason we distribute it in a separate piece of software is that it is

intended to be used by other genome graph tools - not just pandora. For instance it is also

used to provide the input for gramtools

(https://www.biorxiv.org/content/10.1101/2021.02.03.429603v1).

- I think it would be sensible to put make_prg on either/both PyPI and/or conda. Currently the

dependencies are unmanaged, and it installs into the global environment by default.

We now provide a precompiled binary for the version used in the paper and it can also be

installed via pip (see https://github.com/leoisl/make_prg#install). In addition, we have added

an (updated, more stable) version to bioconda

(https://github.com/iqbal-lab-org/make_prg#conda).

- I used the docker image to run pandora. Though the command to pull it is given, no further

information on how to use this image and share data with it was listed. It took a little time to

work this out. I would recommend adding instructions on how to then use the image to the

README.

Additionally, what would you recommend when docker is not an option for pandora (e.g. on

HPC systems)?

We have updated the README to be more specific about how to use the containers for both

docker and singularity (singularity is often an option when docker is not avaliable).

In addition we have now dramatically improved the installation from source and updated the

documentation to better aid this. We now also provide a (Linux) pre-compiled binary

(https://github.com/rmcolq/pandora/#no-installation-needed---precompiled-portable-binary)

and pandora can also be installed through bioconda:

https://github.com/rmcolq/pandora/#conda

- It was difficult to know whether I should be using an unaligned fasta or MSA for each of the

make_prg and pandora steps. It would be useful to highlight this. pandora gives a slightly

uninformative error message if you use an MSA as input.

At present, *make_prg* takes an MSA and creates a PanRG. *Pandora* takes a read file

(unaligned) and the PanRG. We have updated the Usage wiki and will be extending

*make_prg* to accept unaligned sequences in due course. We also added a self-contained

example that shows how to run make_prg and pandora, with and without denovo, in a small

dataset: https://github.com/rmcolq/pandora/tree/master/example .

- When running 'pandora map' does this only accept a single read file? What if reads are in

two forward/reverse files? I concatenated my files, but might be worth addressing this in the

docs.

This is a good point - it does only accept a single read file. We have added this to the FAQ,

and will fix this in the future.

- When I ran 'pandora map' it looked successful, but I didn't get any variants in the VCF

output (using -I --genotype). Could this be expected?

This is hard to answer without knowledge of the PanRG/dataset in question, but we would

be happy to investigate if you wanted to raise an issue on github

- The authors may wish to investigate pybind11 for their planned python->C++ conversion, to

keep the useful python code and not reimplement everything (command line args etc) in

C++. I have previously found this package a joy to use.

Thank you!

- Are Ns disallowed in make_prg input? I got an EncodeError using an alignment with Ns.

We know this error. We currently have a branch which allows Ns, and will be looking to

reincorporate the relevant changes once the new and improved *make_prg* has been

finished. For now, ideally no Ns.

Small edits:

- In the abstract: 'The pragmatic option' -> 'a commonly used pragmatic option'

- p12 l15: 'key deliverable' -> 'main advantage'

These have been fixed

- In the discussion: 'not a production tool'. Can the authors expand on exactly what they

mean by this? Should others not use this software? My impression is that it would be good

for others to start using pandora for testing, but not yet incorporate it into their pipelines as

standard.

We apologise for the ambiguous language. At the time of submission, we were unhappy with

the performance (speed) of pandora, which we thought would be a barrier to adoption. At

that time, using a compute cluster, it was taking 8 hours to update the graph with newly

discovered alleles, and ~30-40 hours to compare the 20 genomes in our data (see the

Performance section of the original submission). Thus our “not a production tool” was

referring to this not being yet ready for people to run on hundreds or thousands of genomes

with the turnaround they currently expect for single-reference tools. However, since then we

have made some performance improvements. First, we noticed that the performance figures

in the submission were with the software compiled for debug, rather than release mode - this

essentially means it was set up to run slowly for debug purposes, an oversight on our part.

Fixing this increased mapping speed by a factor of 5. Secondly, we replaced clustal-omega

with MAFFT. Third, we developed a new algorithm for updating a graph based on de novo

discovered alleles, which could be placed into a graph without needing full reconstruction

from scratch (i.e. no need to reconstruct a full MSA). We also reorganised the

implementation of pandora so it could be run multithreaded on a single computer, instead of

running each per-gene/locus job on a different node of a compute cluster. Put together,

these changes reduce the CPU time required to process 20 samples from 73/88 CPU hours

(for illumina/nanopore) to 9/16 CPU hours, and the elapsed (human perceived) time from

45/65 hours (respectively) to 10/27 hours. The final results are essentially unchanged. On

this basis, we have simply removed the “not a production system” sentence.

- Conclusions: 'to our knowledge, the first solution'. I would advise against claims such as

this - the novelty of this work stands on its own.

Agree - we have removed this

- Methods p22 l31 'log-likelihood-inspired' - please explain what these means and why it was

used.

We apologise for the unhelpful language. We use an approximation to the log likelihood in

order to handle issues we had with comparing very small floating point numbers. Text has

been updated to clarify this.

- I could not distinguish between the colours in fig1a - could an alternative diverging scheme

be used?

We have revised the colour scheme in Figure 1.

- In fig1b I was unable to easily match the colours of genes across the genomes.

Highlighting syntenic blocks of genes between each sample with shaded blocks (as in ACT

or genomeribbon)

Given the nature of the cartoon, we were unable to link syntenic blocks, however we have

made the gene blocks wider and introduced an updated color palette which should make it

easier to scan across the genomes.

Reviewer #2:

The authors describe a novel method, pandora, enabling variant calling using a set of

reference genomes stored in a graph data structure. The manuscript is well written and

describes many smart features of pandora together with evaluations comparing pandora to

single reference variant callers.

In general I think this is an interesting and impressive manuscript. I have, however, a few

remarks:

We thank the reviewer for this feedback!

- the main argument for pandora described in the manuscript is that detecting SNPs in the

core genome is not enough. However, the authors do not provide much proof/examples for

how SNPs in the accessory genome - once identified - can be used to enhance our

understanding of these genomes. As this is not clear to me through the manuscript, the

argument loses its power somehow.

Firstly we would argue that it is very difficult to show that accessory variation is useful whilst

it remains largely inaccessible. A major thesis of this paper is that you need a framework in

which to compare many genomes in order to be able to infer the impact of accessory

variation. Using a reference creates a bias; in some specific cases, this is the right thing to

do - especially when you have a very low diversity set of genomes to compare, in an

outbreak. By making the accessory variation accessible, we hope to enable the community

to explore it in a wide range of situations.

In the Discussion, we list some examples where accessory variation is important

“*Many examples from different species have shown that bacteria are able to leverage this*

*genomic flexibility, adapting to circumstance sometimes by using or losing novel genes*

*acquired horizontally, and at other times by mutation. There are many situations where*

*precise nucleotide-level variants matter in interpreting pan-genomes. Some examples*

*include: compensatory mutations in the chromosome reducing the fitness burden of new*

*plasmids [43–45]; lineage-specific accessory genes with SNP mutations which distinguish*

*carriage from infection [46]; SNPs within accessory drug resistance genes leading to*

*significant differences in antibiograms [47]; and changes in CRISPR spacer arrays showing*

*immediate response to infection [48,49].*”

Which we believe is sufficiently clear.

At least it would have be great to see how the accessory genome SNPs identified in the

E.coli dataset compare to the core genome phylogeny of these strains.

When we infer a phylogeny for a set of genomes based on some loci (here, the core

genome), we implicitly assume these loci have existed together for the full ancestral history

of these genomes. This assumption fails for the accessory genes, which will have been

present in the population for widely differing lengths of time. Comparing an “accessory

genome tree” with that from the core, or somehow comparing accessory SNPs with the core

tree would be confusing and hard to interpret. Both congruence and disagreement would be

prone to misinterpretation, and we fear most of the effect would be driven by population

structure in the dataset. We apologise, but think that doing this, especially for our small

dataset selected purely for diversity, would add confusion to the paper.

- what is the limit on the number of bacterial reference genomes pandora can handle?

Strictly, we do not know, but we have built one from the 10,000 genomes in Horesh et al

(https://www.microbiologyresearch.org/content/journal/mgen/10.1099/mgen.0.000499). The

limiting factor becomes the time taken to run panX/roary/panaroo, or whichever tool is being

used to find orthologs. Since we can go as high as 10,000, we believe this is not a significant

limitation for pandora.

How do I select an appropriate set of reference genomes? I was also wondering if the result

influenced by the diversity of chosen reference genomes?

We include the influence of reference panel selection and diversity in the discussion section.

Ideally they should be representative of the samples you wish to compare. As more

references are included, the amount of “new” material each brings to the pan-genome

“catalogue” will decrease in the moderate to high-frequency part of the gene frequency

spectrum, but rare genes will continue to accumulate. The question of how to sample the

genomes cuts to one of the core challenges with understanding bacterial population

structure. Our advice in the original discussion was:

“we would advocate sampling by geography, host species (if appropriate), lifestyle (e.g.

pathogenic versus commensal) and/or environment.”

Which we have now changed to

“we would advocate sampling by **phylogeny**, geography, host species (if appropriate),

lifestyle (e.g. pathogenic versus commensal) and/or environment.”

- The comparisons with single-reference tools seem to have been carefully designed and are

certainly not easy to do, however the presented comparison in the paper is also not easy to

understand. Figure 6 shows "precision" of the evaluated methods, however I could not find a

definition of precision in the text.

We agree with this, and the previous reviewer, and have worked to improve this in our

updated draft. We have now moved the somewhat irrelevant discussion about

methylation-aware basecalling to the methods section and supplement, have moved 2

panels from a 4-panel figure to the supplement, and now direct the reader more rapidly to

the key results. We have defined precision on line 973 of the Methods: essentially we

measure what proportion of VCF records are correct. This is straightforward for SNPs, but

for longer alleles, it is possible to be partially correct - for example, if the true allele is AACT

but the caller claims it is AGCT, is this correct or incorrect? We measure precision as the

proportion of correct bases - here the precision would be 0.75 as 3 of the 4 bases in the

allele were correct.

- While I understand that graph-based variant calling is hit and new, there are already a few

tools out there that address the problem (VG toolkit? GraphTyper? Paragraph? PanGenie

and Giraffe (admittedly these two only appeared in 2020). It would have been useful to see a

direct comparison with other graph based methods to understand the advantages of

pandora, or at least add a paragraph discussing these other methods in comparison to

pandora.

There are indeed a number of graph genome tools. The vast majority of development in this

field has been dominated by the desire to apply them to human genomes. Showing any

value of graph approaches in human genomics is a challenging task, as human genomes

are not very diverse and harbour almost no hard reference bias. We (ZI) showed in Dilthey

et al 2015 that even in the MHC, arguably the most diverse part of the human genome, you

have to work very hard to show a benefit of using graphs. The focus of these tools has

therefore been to somewhat mirror the approach of normal human genomics: generalise

mapping to graphs, and alignment, and prove that this reduces soft reference bias. This

work dominated the period 2014-2018 roughly, culminating in the first vg, graphtyper and

bayestyper papers. Vg is wedded to a single reference graph which tries to capture the full

range of diversity in the genome, whereas graphtyper and bayestyper simply try to use local

DAGs to incorporate diversity from VCFs or from the reads. Recently, an extension of vg,

called giraffe was published, focussing on accelerating performance and genotyping

structural variants, but still giving results in a VCF based on the reference genome. Thus all

of these methods are fundamentally limited by hard reference bias, and will suffer the same

limitations as more basic approaches, which is why we do not benchmark with them.

PanGenie was mentioned by the reviewer - this is essentially a generalisation to graphs of

phasing and imputation methods based on the Lee and Stephens method that have been

used since HapMap, but is not really comparable to the other tools.

We have modified our introduction as follows

“*The use of graphs as a generalisation of a linear reference is an active and maturing field*

*[12–19]. Much recent graph genome work has gone into showing that genome graphs*

*reduce the impact of soft reference bias on mapping [12,14,15], and on generalising*

*alignment to graphs [16,20]. These methods have almost universally been designed for the*

*human pan-genome, where hard reference bias is a comparatively minor issue compared*

*with bacteria (two human genomes are over 99% alignable, whereas two bacteria of the*

*same species might be 50% alignable). In particular, all current graph methods (e.g. vg [12] ,*

*Giraffe [21], GraphTyper [14,15], paragraph [22], BayesTyper [23]) require a reference*

*genome to be provided in advance to output genetic variants in the standard Variant Call*

*Format (VCF) [24] - thus immediately inheriting a hard bias when applied to bacteria (see*

*Figure 1b). Thus there has not yet been any study (to our knowledge) addressing SNP*

*analysis across a diverse cohort, including more variants that can fit on any single*

*reference.*”

- I think the section on Methylation-aware basecalling is disrupting the evaluation as it

discusses the quality of the read data instead of the actual comparison laid out in the

previous section, maybe move this into appendix?

We agree (as does reviewer 1) and have moved this to the Supplementary Materials

- Figure 7 seems to suggest that while pandora is able to recover more SNPs in the

accessory genome, it also misses SNPs in the core genome (e.g. present in 19 or 20

genomes in the sample) that are detected by snippy and samtools. This should be

addressed in the discussion,

This is indeed true, and we have added the following sentence to the limitations paragraph

of the discussion:

*“There are a number of limitations to this study. Firstly, although pandora achieves a gain of*

*recall in rare variation compared with single-reference tools (at least 12-25k more SNPs in*

*loci present in 2-5 genomes out of 20 depending on choice of tool and reference – a lift of at*

*least 7-15% in recall), this is offset by 11% loss of recall at core SNPs. However, the gain in*

*recall of rare variants will increase both with dataset size (due to the U-shaped gene*

*frequency curve) and if the PanRG is constructed from either a better-sampled input*

*reference-panel, or the dataset itself. By contrast, there is no a priori reason why pandora*

*should miss core SNPs, and this issue will need to be addressed in future work.”*

- maybe the authors want to add a suggestion on when to use pandora and when to rely on

snippy?

Single reference Illumina methods (e.g. snippy) have better precision and recall when

comparing closely related samples (e.g. in an outbreak). When the dataset contains diverse

genomes, or when one does not know in advance how similar the genomes are, *pandora*

improves recall by accessing variation across the pan-genome - notably, different subsets of

genomes have differing core genomes, all of which are accessed immediately by pandora.

The strong graph prior means that *pandora* has better precision and recall than current

Nanopore alternatives even with a more closely related set of genomes. We now discuss

this briefly in the Discussion section (line 508):

*“... The impact of this approach does depend on the dataset under study. We find that, if*

*analysing closely related samples, then single-reference methods provide improved recall*

*compared with pandora. However if analysing more diverse datasets, hard reference bias is*

*a bigger issue for single-reference tools, and pandora offers improved recall.”*

- All analyses in this paper seem to be well documented and reproducible in snakemake

pipelines (awesome!), so I tried to run some code myself. I tried installing pandora from

source code on my server (no docker allowed unfortunately), but ran into errors compiling

gatb (lib and boost installed though). It would be great if there were a few hints on common

compile problems on the GitHub page (if known). I was able to install pandora on my laptop

through docker without problems. I then wanted to try pandora on a set of my genomes, but

realised that the tool building the reference graph only takes MSAs as input. It would be

great if this could be extended to include the alignment step.

I went back to use the hands-on toy example provided on the website and that seemed to

work once I figured out how to use docker properly. I personally would prefer to use pandora

as a source code installation.

Thank you, we are delighted that you attempted to run *pandora* and that you had some

success with the example. We have now much improved the installation from source, most

notably we don’t require any dependencies anymore for compilation, except for a C++11

compiler toolset. We also now provide precompiled (Linux) binaries, which requires no

installation at all, and a bioconda recipe for pandora. We have also updated the

documentation. On many servers where docker is not available, it is possible to use

singularity and we have instructions on how to use this. See

https://github.com/rmcolq/pandora/#installation for these different ways of installing pandora.

We also improved our sample example (see

https://github.com/rmcolq/pandora/tree/master/example) so that it automatically downloads

and run the precompiled binaries so that the user can test pandora without even installing it

or make_prg, and also without having to deal with containers to simply test our tool.

We previously used snakemake to orchestrate our jobs, parallelising many steps per locus,

and therefore needing to run tens of thousands of cluster jobs. However many of these jobs

were extremely fast, and so snakemake was unnecessarily cumbersome. We made some

performance improvements (detailed above, but including moving from clustal-omega to

mafft, adding a new algorithm for updating the PRG with new alleles) and now pandora can

be run on a single machine with a few command-line instructions (essentially: make_prg

from_msa, pandora index, pandora discover, make_prg update and pandora compare).

**2^nd^ round**

**Reviewer 1**

This is a very thorough revision: the authors have improved the presentation of their work, made significant computational and performance enhancements, improved the software, and added their insights into genome graph methods (some of which I found particularly useful – the overview of other methods, and the problems with nanopore sequencing variability).

The authors have comprehensively responded to all of my comments, and I have no further changes to suggest.

**Reviewer 2**

I would like to thank the authors for their extensive reviews and detailed answer to all my previous point which have been addressed appropriately in this revision. I think pandora and this paper are a great piece of work and hope that this concept and all its potential applications described in the paper will be explored further in the future.

I only have one very minor suggestion for this review: Figures 7 and 8 would improve if a color legend is directly included (so the reader does not have to scroll back to figure 4 for the corresponding phylogroups). Figure 7 is also missing a y-axis label.
